# Effect of dietary resveratrol on placental function and reproductive performance of late pregnancy sows

**DOI:** 10.3389/fnut.2022.1001031

**Published:** 2022-10-27

**Authors:** Ruizhi Hu, Jijun Tan, Zhanfeng Li, Long Wang, Mingkun Shi, Baizhen Li, Ming Liu, Xupeng Yuan, Jianhua He, Xiaosong Wu

**Affiliations:** ^1^Hunan Collaborative Innovation Center for Utilization of Botanical Functional Ingredients, College of Animal Science and Technology, Hunan Agricultural University, Changsha, China; ^2^Animal Science and Technology College, Beijing University of Agriculture, Beijing, China; ^3^Hunan Xinguang'an Agricultural Husbandry Co., Ltd., Changsha, China

**Keywords:** resveratrol, sow, reproductive performance, placental function, gut microbiota

## Abstract

Placental function is vital to the fetal growth of sows, and resveratrol (RES) can protect cells against oxidative stress, which is one of the major factors impairing placental function. This study aimed to investigate the effect of dietary resveratrol (RES) on placental function and reproductive performance during late pregnancy in a sow model from the aspects of oxidative stress, insulin resistance, and gut microbiota. A total of 26 hybrid pregnant sows (Landrace × Yorkshire) with similar parity were randomly allocated into two groups (*n* = 13) and fed with a basal diet or a diet containing 200 mg/kg of resveratrol from day 85 of gestation until parturition. The dietary supplementation of RES increased the litter weight at parturition by 12.53% (*p* = 0.145), with ameliorated insulin resistance (HOMA-IR), increased triglyceride (TG) levels, and decreased interleukin (IL)-1β and IL-6 levels in serum (*p* < 0.05). Moreover, resveratrol increased the placental vascular density (*p* < 0.05) with the enhanced expression of nutrient transporter genes (*SLC2A1* and *SLC2A3*) and antioxidant genes, such as *superoxide dismutase 2* (*SOD2*) and *heme oxygenase-1* (*HO-1*) but declined the expression of inflammatory genes, such as *IL-1*β and *IL-6* (*p* < 0.05). The characterization of the fecal microbiota revealed that resveratrol decreased the relative abundance of the *Christensensllaceae R-7 group* and *Ruminococcaceae UCG-008* (*p* < 0.05), which had a positive linear correlation with the expression of *IL-1*β and *IL-6* (*p* < 0.05), but had a negative linear correlation with the expression of *SOD2, HO-1, SLC2A1*, and *SCL2A3* genes (*p* < 0.05). These data demonstrated that dietary supplementation with resveratrol can improve placental function with ameliorated insulin resistance, oxidative stress, and inflammation potentially by regulating *Ruminococcaceae UCG-008* and the *Christensensllaceae R-7 group* in sows.

## Introduction

Placental function is vital to the nutrition supply of a fetus and thus has a strong impact on the reproductive performance of humans and animals. Over the past 20 decades, genetic improvement has increased the productivity of the sow herd, and nowadays high-yielding sows achieve 35 pigs per sow per year (1). However, an increase in the litter size results in a significant decrease in piglet birth weight (2) and gives piglets a harder time accessing the mammary glands and consuming less colostrum, and leads to higher pre-weaning mortality rates (3). Piglets born at a low birth weight have fewer glycogen reserves right after birth, making them more vulnerable to hypothermia and hypoglycemia within the first 24 h of life (4, 5).

Late pregnancy is a crucial period for fetal growth, and the maternal body undergoes substantial metabolic changes (6, 7), which make sows adapt from anabolic to catabolic metabolism (8). Increased metabolic intensity may lead to low-level inflammation (9), progressive oxidative stress (10), and insulin resistance (11). Meanwhile, the gut microbiota undergoes dramatic remodeling during late pregnancy that can affect a sow's physiological state and metabolic process (12). Our previous studies have demonstrated that nutritional additives can improve antioxidant capacity and reduce inflammation by modulating the intestinal flora in late pregnancy (13, 14). Moreover, abnormal glucose metabolism can decrease glucose utilization and placental nutrient transport to cause a low-weight maternal fetus (15), and increased systemic oxidative stress in late gestation can result in vascular dysfunction in the placenta (16, 17). These studies suggest that alleviating inflammation, oxidative stress, and insulin resistance in late gestation might be a promising strategy for improving placental function and increasing the reproductive performance of sows.

The dietary supplementation of phytochemicals during pregnancy can positively affect reproductive performance in sows (18). Polyphenols are plant-derived natural bioactive compounds with a strong antioxidant ability (19), and our previous studies have shown that polyphenols possess biological functions, such as anti-inflammatory, antioxidant, and alleviation of insulin resistance (20, 21). Resveratrol (RES) is a polyphenol that belongs to the stilbene family of phytoalexins and has shown an effect against oxidative stress and apoptosis in embryos of diabetic dams (22). In addition, it has been reported that RES may increase uterine artery blood flow velocity and fetal weight in murine models (23), and the biological activity of RES appears to be closely linked with modulating gut microbiota (24). Therefore, this study aimed to investigate the effect of dietary RES on the placental function and reproductive performance of sows during late pregnancy from the aspects of oxidative stress, insulin resistance, and gut microbiota.

## Materials and methods

The animal experiment protocol used for the present study was approved by the Hunan Agricultural University Institutional Animal Care and Use Committee.

### Materials and reagents

Resveratrol (≥98%) was provided by Hunan Engineering and Technology Center for Natural Products. Ingredients for the basal diet were provided by Hunan Xinguang'an Agricultural Husbandry Co., Ltd. (Changsha, Hunan, China).

### Experimental design and diets

This study was carried out on the farm of Hunan Xinguang'an Agricultural Husbandry Co., Ltd. A total of 26 pregnant sows (Landrace × Yorkshire) with similar body condition scores and parity from 2 to 5 were used in this study. Sows were randomly divided into two treatments (*n* = 13), housed individually in gestation stalls, and had free access to water under environmental temperature. The experiment started on the 90th day of gestation and continued until delivery. Based on our pilot experiment, sows in the control group (CTL) were fed a basal diet, while sows in the RES group were fed a basal diet containing 200 mg/kg of RES based on our pilot experiment (RES intake 640 mg/day/sow). Approximately 3.2 kg of feed/sow/day was fed at 6:00 a.m., 12:00 p.m., and 6:00 p.m. The experiment started on day 85 of gestation and lasted until delivery. The composition of the basal diet, which meets the nutritional requirements of pigs according to NRC (2012), as is shown in Supplementary Table S1.

### Measurements of reproductive performance and sample collection

After delivery, the number of live births, litter weight at parturition, and average weight of piglets born alive were measured immediately. On the day of parturition, blood samples (5 ml) of sows were collected from the marginal auricular vein into anticoagulant-free vacuum tubes and centrifuged on 1,500 × g for 10 min after standing at room temperature for 30 min to obtain serum. The placentae were collected after being weighed and snap-frozen in liquid nitrogen (3–4 cm from the cord insertion point). Fecal samples (~2 g from each sow) were collected into sterile tubes after defecation in the morning, and snap-frozen in liquid nitrogen. Finally, these samples were stored at −80°C until further analysis. Another about 1 cm × 1 cm fresh placentas tissues were immediately fixed in 4% paraformaldehyde (G1119, Servicebio Technology Co., Ltd. Wuhan, Hubei, China) for at least 24 h for H&E staining.

### Histomorphological analysis

Each sow's placentae were sectioned for histological analysis as in a previous study (14). Briefly, placenta tissues were fixed in 4% paraformaldehyde overnight and submitted to Wuhan Servicebio Technology Co., Ltd (Wuhan, Hubei, China) for paraffin embedding and sectioning, then put the slices into xylene for 20 min two times, 75% alcohol for 5 min, and washed with tap water. Then, the slices were stained with hematoxylin dye solution for 3–5 min, washed with tap water, differentiated with differentiation solution, washed with tap water, H&E stain returned to blue, and rinsed with tap water. The slices were dehydrated with 85 and 95% gradient alcohol for 5 min and stained with eosin for 5 min. Then, put in absolute ethanol I for 5 min, absolute ethanol II for 5 min, absolute ethanol III for 5 min, dimethyl I for 5 min, and xylene II for 5 min, transparent and sealed with neutral gum. For each slice, the total number of vessels in the placental stroma areas was determined, and then corrected with the total placental stroma areas measured (per unit area as mm^2^).

### Measurement of serum biochemical indices

The activity of total superoxide dismutase (T-SOD) and serum levels of thiobarbituric acid reactive substances (TBARS), glucose (GLU), total cholesterol (TC), triglycerides (TGs), high-density lipoproteins (HDL-c), and low-density lipoprotein cholesterol (LDL-c) were determined by using respective assay kits (Nanjing Jiancheng Bioengineering Institute, Nanjing, China) according to the manufacturer's instructions as described previously.

Serum levels of hormones, such as progesterone (PRO), follicle-stimulating hormone (FSH), and prolactin (PRL), insulin, and serum cytokines, such as interleukin (IL)-1β, IL-6, and Monocyte Chemotactic Protein 1 (MCP1) were measured with respective ELISA kits (Aifang biological Co., Ltd, Changsha, Hunan, China) according to the manufacturer's manual.

Insulin resistance and sensitivity were evaluated by the homeostasis model assessment (HOMA) values using the following indirect methods.


$$\begin{array}{l} \;HOMA-insulin\;resistance\;(HOMA-IR)\\ =\;[Fasting\;insulin\;(mIU/L)\;\times \;Fasting\;glucose\;(mmol/L)]/22.5\\ \;HOMA-insulin\;sensitivity\;(HOMA-IS)\\ =\;1/[Fasting\;insulin\;(mIU/L)\;\times Fasting\;glucose\;(mmol/L)] \end{array}$$

### Real-time PCR

The quantitative PCR assays were validated according to the MIQE guidelines (25). Total RNA from the placenta or PVECs was extracted with the Total RNA Kit (Steadypure universal RNA extraction kit, Accurate Biotechnology Co., Ltd., Changsha, Hunan, China) according to the manufacturer's instructions. The concentration of RNA was quantified using a NanoDrop^®^ lite (Thermo Fisher, USA). Reverse transcription of 1 μg total RNA was performed by a reverse transcription kit (Evo M-MLV RT Premix, Accurate Biotechnology Co., Ltd., Changsha, Hunan, China). The PCR reactions were performed in a 20 μl total reaction volume, which included 10 μl of 2 × SybrGreen qPCR Master Mix (SYBR^®^ Green Premix Pro Taq HS qPCR Kit), 0.4 μl of each of the forward and reverse primers (10 μmol/L), 2 μl of cDNA template, and 7.2 μl of sterilized water. The PCR was carried out on a LightCycler480 Real-Time PCR system (Rotkreuz, Switzerland). The thermal cycler parameters were as follows: 3 min at 95°C, 40 cycles for 5 s at 95°C, and 30 s at 60°C. The stability of the β*-actin* and *GADPH* genes was evaluated by measuring the fluctuation range of the Ct values. The 2^−ΔΔCT^ method was used for data analysis. Primers used in this study are shown in Supplementary Table S2.

### Characterization of the gut microbiota

The fecal microbiota was characterized by 16S rDNA gene sequencing as described previously (14). Briefly, total DNA was extracted from fecal samples (six random samples from each group) by using a DNA Isolation Kit (MoBio Laboratories, Carlsbad, CA, USA) following the manufacturer's manual. The V3-4 hypervariable region of the bacterial 16S rRNA gene was amplified with the primers 338F (5′-ACTCCTACGGGAGGCAGCA-3′) and 806R (5′-GGACTACHVGGGTWTCTAAT-3′). The PCR was carried out on a Mastercycler Gradient (Eppendorf, Germany) using 25 μl reaction volumes, containing 12.5 μl KAPA 2G Robust Hot Start Ready Mix, 1 μl Forward Primer (5 μmol/L), 1 μl Reverse Primer (5 μmol/L), 5 μl DNA (total template quantity is 30 ng), and 5.5 μl H_2_O. Cycling parameters were 95°C for 5 min, followed by 28 cycles of 95°C for 45 s, 55°C for 50 s, and 72°C for 45 s with a final extension at 72°C for 10 min. Three PCR products per sample were pooled to mitigate reaction-level PCR biases. The PCR products were purified using a QIAquick Gel Extraction Kit (QIAGEN, Germany), quantified using Real-Time PCR, and sequenced on the Miseq platform at Allwegene Technology Inc., Beijing, China. Qualified reads were separated using the sample-specific barcode sequences and trimmed with Illumina Analysis Pipeline Version 2.6. The dataset was analyzed using QIIME (Version 1.8.0). The sequences were clustered into operational taxonomic units (OTUs) at a similarity level of 97%, to generate rarefaction curves and to calculate the richness and diversity index. The Ribosomal Database Project (RDP) Classifier tool was used to classify all sequences into different taxonomic groups.

### Statistical analysis

The significant differences between groups were analyzed by a *t*-test with the SPSS 23.0 program (SPSS 23, IBM Corp., Armonk, NY, USA). The Pearson correlation and linear regression methods were used for the correlation analysis between the abundance of the top 50 microbial genera and placental gene expression. The value of *p* < 0.05 was considered significant.

## Results

### The impact of RES on the reproductive performance of sows

As shown in Table 1, there was no significant difference in total born number, the number of live birth, weak piglets' number, litter weight at parturition, and the average weight of piglets born alive. However, dietary supplementation of RES increased the litter weight at parturition by 12.53% (*p* = 0.145).

**Table 1:** The effect of RES on the reproductive performance of sows.

**Item**	**CTL**	**RES**	***P-*value**
Total born number	14.75 ± 3.22	15.00 ± 4.18	0.866
The number of live birth	12.25 ± 3.27	13.27 ± 3.44	0.445
Litter weight at parturition, kg	16.06 ± 3.91	18.36 ± 3.87	0.145
Average weight of piglets born alive, kg	1.33 ± 0.17	1.42 ± 0.24	0.271
Weak piglets' number	0.54 ± 0.88	0.08 ± 0.27	0.083
Stillborn piglets' number	2.50 ± 2.95	1.72 ± 1.58	0.415

CTL, a basal diet; RES, a basal diet containing 200 mg/kg, weak piglets defined as below 20% of the average weight of piglets, RES, n = 13.

### The effect of RES on insulin resistance and lipid metabolism in perinatal sows

**Figure 1:**
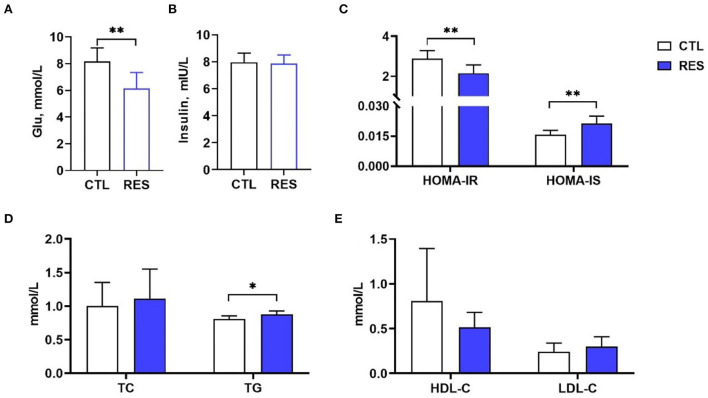
The effect of RES on glucose metabolism and lipid metabolism in perinatal sows. **(A)** Glu, **(B)** insulin, **(C)** HOMA-IR and HOMA-IS, **(D)** TC and TG, and **(E)** HDL-C and LDL-C. Glu, glucose; HOMA-IR, homeostasis model assessment-insulin resistance; HOMA-IS, homeostasis model assessment-insulin sensitivity; TC, total cholesterol; TG, triglycerides; HDL-C, high-density lipoprotein cholesterol; LDL-C, low-density lipoprotein cholesterol; CTL, a basal diet; and RES, a basal diet containing 200 mg/kg resveratrol. Data are expressed as mean ± SD (*n* = 10); *, *p* < 0.05; and **, *p* < 0.01.

As shown in Figure 1, RES significantly decreased the serum glucose (Figure 1A) in perinatal sows (*p* < 0.01) but showed no influence on the insulin level (Figure 1B). HOMA-IR and HOMA-IS were used to reflect insulin resistance and insulin sensitivity, respectively. Supplementation of the diet with RES significantly decreased HOMA-IR and increased HOMA-IS (Figure 1C) (*p* < 0.01). Moreover, the TG (Figure 1D) in the RES group was significantly increased compared with the CTL group (*p* < 0.05), while there was no significant change in TC, HDL-C, and LDL-C.

### The effect of RES on serum cytokines and hormones in perinatal sows

**Figure 2:**
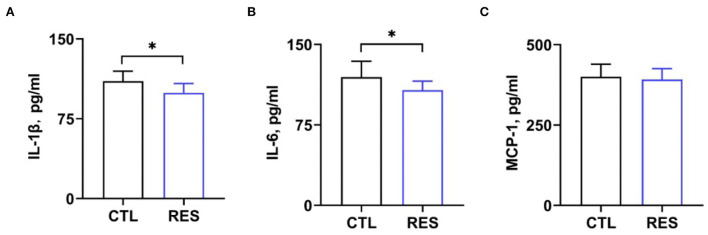
Effects of RES on blood inflammatory markers. Serum levels of **(A)** IL-1β, **(B)** IL-6, and **(C)** MCP-1 were measured by using ELISA kits. CTL, a basal diet; and RES, a basal diet containing 200 mg/kg resveratrol. Data are expressed as mean ± SD (*n* = 10); *, *p* < 0.05.

**Figure 3:**
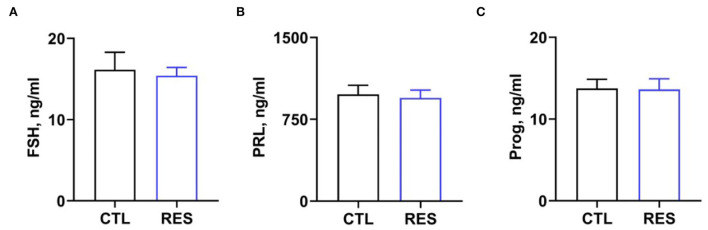
The effect of resveratrol on serum hormone levels in perinatal sows. **(A)** Follicle-stimulating hormone (FSH), **(B)** prolactin, and **(C)** progesterone. CTL, a basal diet; and RES, a basal diet containing 200 mg/kg resveratrol. Data are expressed as mean ± SD (*n* = 10); *, *p* < 0.05.

Serum cytokine and hormone levels in perinatal sows were measured to assess the inflammatory status in perinatal sows. Figure 2 shows that RES decreased serum levels of IL-1β (Figure 2A) and IL-6 (Figure 2B) (*p* < 0.05). However, there was no significant difference in the levels of MCP-1. Additionally, RES shows no significant effects on serum levels of FSH, prolactin, and progesterone (Figure 3).

### Effects of RES on vessel density and the expression of genes regulating nutrient transport, antioxidant defense, and inflammation in placenta

**Figure 4:**
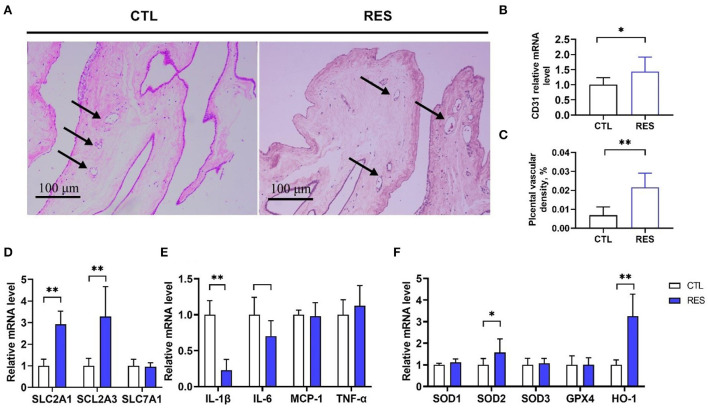
The effect of RES on vessel density, nutrient transport, antioxidant, and inflammation mRNA expression in the placental. **(A,B)** The hematoxylin and eosin method was used to examine blood vessel density in placental tissues, and the black arrows indicate placental blood vessels (bar = 100 μm, *n* = 12); **(C)** *CD31* mRNA expression; **(D)** Nutrient transporters *SLC2A1, SCL2A3*, and *SLC7A1* genes expression; **(E)** Inflammatory factors *IL-1*β, *IL-6, MCP1*, and *TNF-*α genes expression; **(F)** Antioxidant genes *SOD1, SOD2, SOD3, GPX4*, and *HO-1* expression. CTL, a basal diet; and RES, a basal diet containing 200 mg/kg resveratrol. Data are expressed as mean ± SD (*n* = 8); *, *p* < 0.05; and **, *p* < 0.01.

As shown in Figure 4, the placenta in the RES group showed significantly higher (*p* < 0.01) blood vessel density (Figures 4A,B) and the *CD31* messenger RNA (mRNA) expression (a biomarker of the endothelial cell in small vessels) (Figure 4C). Meanwhile, RES significantly increased the expression of nutrient transporter genes of *SLC2A1* and *SCL2A3* (*p* < 0.01) but showed no effect on *SLC7A1*. Moreover, the expression of *IL-1*β and *IL-6* were downregulated by RES, and the expression of *SOD2* and *HO-1* were upregulated by RES in the placenta (*p* < 0.05).

### The effect of RES on the gut microbiota

**Figure 5:**
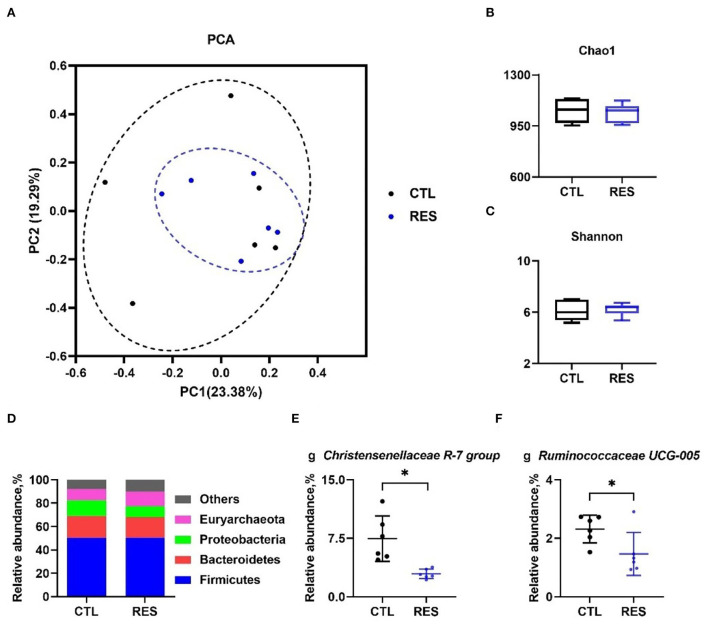
Modulation of the gut microbiota by RES. The effect of RES on genus-based (*n* = 6) principal component analysis (PCA) **(A)**, the Chao 1 index **(B)**, the Shannon index **(C)**, the effect of RES on the relative abundance of main microbes at the phylum level **(D)** and the genera *Christensenellaceae R-7 group* **(E)**, and *Ruminococcaceae UCG-005* **(F)**. CTL, a basal diet; and RES, a basal diet containing 200 mg/kg resveratrol. Data are expressed as mean ± SD (*n* = 6); and *, *p* < 0.05.

As shown in Figure 5, principal component analysis (PCA) indicated that the CTL and RES groups had a similarity cluster (Figure 5A), and RES showed limited effects on the Chao 1 index (Figure 5B) and the Shannon index (Figure 5C). Firmicutes, Bacteroidetes, Proteobacteria, and Euryarchaeota accounted for 90% of the total microbes at the phylum level (Figure 5D), and RES significantly downregulated the relative abundances of the *Christensenellaceae R-7 group* (Figure 5E), and *Ruminococcaceae UCG-005* (Figure 5F) at the genus level (*p* < 0.05).

### Correlation analysis of the gut microbiota and placental gene expression

**Figure 6:**
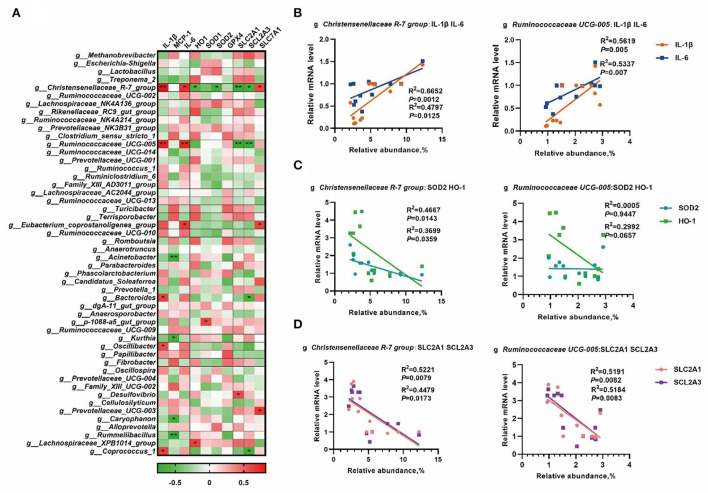
The correlation analysis of the gut microbiota and placental gene expression. The correlation analysis between the abundance of the top 50 microbial genera and placental gene expression by Spearman's correlation analysis **(A)**, linear regression analyses between genera and placental mRNA expression of inflammatory cytokines **(B)**, mRNA expression of antioxidative genes **(C)**, and expression of genes involved in the regulation of nutrient transport in placenta **(D)**. *, *p* < 0.05; and **, *p* < 0.01.

To further understand the role of whole gut microbiota in regulating oxidative and inflammatory status, the abundance of the top 50 genera was chosen to process correlation analysis with placental gene expression to better understand the role of the whole gut microbiota in regulating oxidative and inflammatory status. As shown in Figure 6A, a total number of 14 microbial genera were significantly correlated (*p* < 0.05) with the placental nutrient transport, antioxidant defense, and inflammation-related mRNA expression. Based on the results in Figure 5, the *Christensenellaceae R-7* group and *Ruminococcaceae UCG-005* were selected to perform linear regression analyses. As shown in Figure 6B, the *Christensenellaceae R-7 group* and *Ruminococcaceae UCG-005* had a positive linear correlation with the expression of *IL-1*β and *IL-6* (*p* < 0.05). Moreover, the *Christensenellaceae R-7 group* had a negative linear correlation with *SOD2* and *HO-1* (*p* < 0.05) (Figure 6C). In addition, the *Christensenellaceae R-7 group* and *Ruminococcaceae UCG-005* had a negative linear correlation with *SLC2A1* and *SCL2A3* (*p* < 0.05) (Figure 6D).

## Discussion

The latter part of pregnancy is critical for fetal development and the critical period of litter weight at parturition. About 60% of fetal body weight gain occurs in late gestation (6). During pregnancy, sows have a complex and dynamic metabolism, which includes nutrient intake, digestion, and absorption, and as well as complex transport processes, such as nutrients transported from maternal tissues to fetal and breast tissues (8). As a result, the metabolic intensity in the sow is significantly increased during late pregnancy due to the rapid growth of the fetus. Studies have shown that sows suffered increased oxidative stress during delivery of late gestation, as indicated by their increased reactive oxygen (ROS), 8-hydroxy-deoxyguanosine, and thiobarbituric acid reactive substance levels (16). A series of metabolic changes characterize the metabolic syndrome in late gestation, especially insulin resistance. Yang et al. (15) have reported that starch inclusion in the maternal diet can improve the sows' insulin resistance during late gestation, increase piglet weight at birth and weaning, and decrease the stillbirth rate.

In the present study, supplementation of 200 mg/kg RES in late gestation can be increased, (although not significantly, *p* > 0.05). An increase in insulin resistance during gestation has been considered as a fundamental cause of the reduced reproductive performance of sows (26). Previous studies have demonstrated that RES may attenuate insulin resistance to improve glucose homeostasis and metabolic disorders (27, 28). Our results showed that RES improved insulin resistance, which suggests that RES may improve metabolic disorders and maternal metabolism during late gestation. The development of insulin resistance during pregnancy is considered to be induced by some placental hormones (29). For insulin-secreting beta-cells that reside in the pancreas, maternal prolactin can directly promote their proliferation, enhance their sensitivity, and increase insulin secretion from this group of cells after glucose stimulation (29). Progesterone could induce insulin resistance by inhibiting GLUT-4 translocation, decreasing the expression of insulin receptor substrate-1 (IRS-1), and blocking the glucose absorption by adipocytes (30). Moreover, FSH has been reported to have a negative association with insulin resistance (31). However, RES showed no significant effect on these hormones in the present study, indicating that RES may improve insulin resistance through other mechanisms, rather than regulating hormones level.

The placenta is a highly vascularized tissue and is essential for the growth and development of the fetus. The development of the epitheliochorial placenta of pigs begins at the embryonic stage (29). It has been reported that the piglet birth weight has a significant positive correlation with placental vascular density (17), and RES may increase uterine artery blood flow and fetal weight (32). In this study, RES increased the placental vessel density with upregulated mRNA expression of *CD31*, which is a biomarker of endothelial cells in blood vessels (33). Meanwhile, RES increased the mRNA levels of *SLC2A3* and *SLC2A1*, which code glucose transporter GLUT-3 and GLUT-1, respectively. However, RES showed no significant effect on *SLC7A1*, which encodes a cationic amino acid transporter (34). Thus, RES might benefit fetal growth and development by improving placental glucose transport, which is the primary energy source for fetal development (35). Oxidative stress and inflammation have been considered important factors that affect the growth and function of the placenta. The activation of the NF-κB pathway in the placental has been reported to have a close relationship with the downregulation of angiogenesis-related genes, such as *HIF1*α, *VEGFA*, and *ANGPTL6* (36). Oxidative stress can also induce vascular endothelial injury and abnormal development of the placenta vascular (17). RES is a known antioxidant and anti-inflammatory agent and has been proved to promote the nuclear accumulation, DNA binding, and transcriptional activity of Nrf2 (37) in sows' placenta. Moreover, RES can suppress Cd-induced apoptosis in the placenta and JEG-3 cells and decrease Cd-induced expression of TNF-α, IFN-γ, MCP-1, MIP-2, and KC in the placenta (32). In this study, RES alleviated placenta oxidative stress and inflammation *via* improving *SOD2* and *HO-1* expression, but inhibiting IL-1β and IL-6 production.

Increasing pieces of evidence have suggested that the gut microbiota plays a crucial role in pregnancy, and maternal gut microbiota undergoes dramatic changes throughout the gestation period. The abundance and diversity of the gut microbiota increase as the pregnancy continues and they are closely related with the metabolism and reproduction of the host (38). Our previous studies have shown that the gut microbiota is associated with low-grade inflammation and oxidative stress in normal pregnancy, and β-carotene can inhibit the production of inflammatory cytokines and improve antioxidant capacity by modulating the gut microbiota (13, 14). RES is a kind of polyphenol with low *in vivo* bioavailability (39), and it can interact with the gut microbiota in the hindgut (40). When passing the small intestinal lumen, RES can be absorbed into the enterocyte and undergoes sulfation and glucuronidation. The RES and conjugated metabolites that exit from the apical membrane of the small intestine will move to the large intestine and further be metabolized by the gut microbiota to generate dihydroresveratrol, lunularin, and 3,4'-dihydroxy-trans-stilbene (41, 42). Previous studies have revealed that the biological functions of RES may be attributed to the regulation of gut microbiota (43, 44). In the present study, dietary supplementation of RES mainly downregulated the relative abundance of *Ruminococcaceae UCG-008* and the *Christensensllaceae R-7 group* at the genus level. *Ruminococcaceae UCG-008* belongs to the family of *Ruminococcaceae* and has a close correlation with butyrate production (45), and negatively correlated with inflammatory cytokines in our previous study (46). The *Christensensllaceae R-7 group* belongs to the family of *Christensensllaceae*, which has a negative correlation with lipid biosynthesis and energy metabolism pathways in humans (47). In the present study, the *Christensensllaceae R-7 group* and *Ruminococcaceae UCG-008* had a positive linear correlation with *IL-1*β and *IL-6* but had a negative linear correlation with *SLC2A1* and *SCL2A3*. However, only the *Christensensllaceae R-7 group* had a negative linear correlation with *SOD2* and *HO-1*. These results suggest that the *Christensensllaceae R-7 group* and *Ruminococcaceae UCG-008* may play an important role in the beneficial effects of RES on placental function.

## Conclusion

Dietary supplementation of resveratrol during late pregnancy showed a positive effect on the placental function of sows, with ameliorated insulin resistance (HOMA-IR), increased triglyceride (TG) levels, and reduced levels of inflammatory cytokines, such as IL-1β and IL-6. Resveratrol could enhance the placental vascular density by upregulating the expression of nutrient transporters genes (*SLC2A1* and *SLC2A3*), attenuate placental oxidative stress and inflammation by promoting the expression of antioxidant genes (*SOD2* and *HO-1*), and suppressing inflammatory genes (*IL-1*β and *IL-6*). An analysis of the gut microbiota revealed that resveratrol can reduce the relative abundance of the *Christensensllaceae R-7 group* and *Ruminococcaceae UCG-008*, which have a positive linear correlation with IL-1β, IL-6, SLC2A1, and SCL2A3.

## Data availability statement

The original contributions presented in the study are publicly available. This data can be found here https://www.ncbi.nlm.nih.gov/bioproject/PRJNA885401.

## Ethics statement

The animal study was reviewed and approved by Hunan Agricultural University Institutional Animal Care and Use Committee.

## Author contributions

RH is the primary investigator in this study. JT and ZL participated in the animal experiments. LW and MS performed statistical data analysis. BL, ML, and XY participated in the sample analysis. JH and XW designed this study and wrote the manuscript as the corresponding author. All authors contributed to the article and approved the submitted version.

## Funding

The authors gratefully acknowledge the support from the National Natural Science Foundation of China (U22A20515; 31772819) and the earmarked fund for CARS (CARS-35).

## Conflict of interest

Author XY was employed by Hunan Xinguang'an Agricultural Husbandry Co., Ltd. The remaining authors declare that the research was conducted in the absence of any commercial or financial relationships that could be construed as a potential conflict of interest.

## Publisher's note

All claims expressed in this article are solely those of the authors and do not necessarily represent those of their affiliated organizations, or those of the publisher, the editors and the reviewers. Any product that may be evaluated in this article, or claim that may be made by its manufacturer, is not guaranteed or endorsed by the publisher.
