# Supplementary material for: Effect of dietary resveratrol on placental function and reproductive performance of late pregnancy sows
Source: Front Nutr. 2022 Oct 27;9:1001031. doi: 10.3389/fnut.2022.1001031 (PMC9673905; doi:10.3389/fnut.2022.1001031)
Supplement: Supplementary file 1 [file Data_Sheet_1.docx]

**Supplementary Material**

**Supplemental Table 1.** Composition of the basal diet

| Ingredients | % |
| --- | --- |
| Corn | 56.9 |
| Rice bran | 8 |
| Soybean meal (CP 46%) | 12.5 |
| Soy oil | 1 |
| Soybean hull (CP 10%) | 18 |
| NaCl | 0.45 |
| Choline chloride (50%) | 0.16 |
| Limestone | 1.2 |
| CaHPO_4_ | 1.15 |
| NaHCO₃ | 0.3 |
| Lys，% | 0.1 |
| Thr，% | 0 |
| Vitamin Premix | 0.04 |
| Mineral Premix | 0.1 |
| Mildew Preventive | 0.1 |
| Total* | 100 |
| Calculated nutrient composition |  |
| DE，Mcal/kg | 3.07 |
| CP，% | 12.7 |
| Ca，% | 0.87 |
| Total P，% | 0.6 |
| Available P，% | 0.4 |
| Lys，% | 0.72 |
| Met，% | 0.22 |
| Thr，% | 0.52 |

The vitamin premix provided for per kg of feed: VA, 9920 IU; VD3, 1985 IU; VE, 66 IU; VK 4.4 mg; VB3, 44 mg; VB2, 10 mg; VB5, 33 mg; VB12, 37 mg; VB7, 220 mg; VB9, 1325 mg; VB1, 2.2 mg; VB6, 3.3 mg. The mineral premix provided for per kg of feed: Cu, 8 mg; Fe, 80 mg; Mn, 45 mg; Zn, 80 mg; I, 0.5 mg; Se, 0.3 mg.

**Supplemental Table 2.** GenBank accession numbers, sequences of forward and reverse primers, and fragment sizes used for Real-Time PCR

| Target | GenBank number | Primer sequence | Size, bp |
| --- | --- | --- | --- |
| *SLC2A3* | XM 021092391.1 | F:5' TCTCCATCATGCTCCAGCTCTCC 3'  R: 5' GGCATAGATTGGCTCCTGAACACC 3' | 107 |
| *SLC2A1* | XM 021096908.1 | F:5' GCTCCTGGTCCTGTTCTTCATCTTC 3'  R:5' CTCGGGTGTCTTGTCGCTTTGG 3' | 124 |
| *SLC7A1* | NM 001012613.1 | F:5' AACCCAGACATCTTTGCCGTGATC 3'  R:5' CCATTATGAAGCCCAGGACCAGAA 3' | 130 |
| *CD31* | NM 213907.1 | F:5' GGTGGTCAAGAGAAGCAATGAGGTC 3'  R:5' AAATGGGCGAGGTTCCGTTTATGG 3' | 146 |
| *IL-1β* | NM 214055.1 | F:5'AAGAGGGACATGGAGAAGCGATTTG 3'  R:5' TTGTTCTGCTTGAGAGGTGCTGATG 3' | 114 |
| *IL-6* | NM 214399.1 | F:5'GAGATTGGCATGGCTTTATTTG 3'  R:5'ACTGCTGTCACCTTCACCGTT 3' | 93 |
| *TNF-α* | NM 214022.1 | F:5' GCACTGAGAGCATGATCCGAGAC 3'  R:5' CGACCAGGAGGAAGGAGAAGAGG 3' | 120 |
| *MCP-1* | NM 214214.1 | F:5' TCTCCAGTCACCTGCTGCTATACAC 3'  R:5' CTGCTTCTTTAGGACACTTGCTTGC 3' | 104 |
| *SOD1* | NM 001190422.1 | F:5' CAGGTGCAGGTCCTCACTTCAATC 3'  R:5' GTCACATTGCCCAGGTCTCCAAC 3' | 91 |
| *SOD2* | NM 214127.2 | F:5' TTCTGGACAAATCTGAGCCCTAACG 3'  R:5' CGACGGATACAGCGGTCAACTTC 3' | 122 |
| *SOD3* | NM 001078688.1 | F:5' GCTGCTCTGTGCTTACCTGCTC 3'  R:5' CTGCGTCAACTCCTGCCAGATC 3' | 145 |
| *GPX4* | NM 214407.1 | F:5' GGCACATGGTGAACCTGGACAAG 3'  R:5' TCCGTAAACCACACTCAGCATATCG 3' | 138 |
| *HO1* | NM 001004027.1 | F:5' CCAGGTCCTCAAGAAGATTGCTCAG 3'  R:5' GGTCATCTCCCAGAGTGTTCATTCG 3' | 145 |
| *Beta actin* | XM 003124280.5 | F:5' TTGATGTCCCGCACGATC 3'  R:5' CCTGAACCCCAAAGCCA 3' | 182 |
| *GAPDH* | NM 001206359.1 | F:5' TGAAGGTCGGAGTGAACGGA 3'  R:5' TGGGTGGAATCATACTGGAACA 3' | 148 |
